# Structural insight into nucleotide recognition by human death-associated protein kinase

**DOI:** 10.1107/S0907444908043679

**Published:** 2009-02-20

**Authors:** Laurie K. McNamara, D. Martin Watterson, Joseph S. Brunzelle

**Affiliations:** a Center for Drug Discovery and Chemical Biology, Northwestern University, Chicago, Illinois 60611, USA; b Department of Molecular Pharmacology and Biological Chemistry, Northwestern University Feinberg School of Medicine, USA; c Life Sciences Collaborative Access Team, Advanced Photon Source, Argonne, Illinois 60439, USA

**Keywords:** death-associated protein kinase, serine/threonine protein kinases, nucleotides, glycine-rich loops, Ca^2+^/calmodulin-regulated protein kinases, apoptosis, brain

## Abstract

Death-associated protein kinase (DAPK) is a member of the Ca^2+^/calmodulin-regulated family of serine/threonine protein kinases. The role of the kinase activity of DAPK in eukaryotic cell apoptosis and the ability of bioavailable DAPK inhibitors to rescue neuronal death after brain injury have made it a drug-discovery target for neurodegenerative disorders. In order to understand the recognition of nucleotides by DAPK and to gain insight into DAPK catalysis, the crystal structure of human DAPK was solved in complex with ADP and Mg^2+^ at 1.85 Å resolution. ADP is a product of the kinase reaction and product release is considered to be the rate-limiting step of protein kinase catalytic cycles. The structure of DAPK–ADP–Mg^2+^ was compared with a newly determined DAPK–AMP-PNP–Mg^2+^ structure and the previously determined apo DAPK structure (PDB code 1jks). The comparison shows that nucleotide-induced changes are localized to the glycine-rich loop region of DAPK.

## Abstract 2

The crystal structures of DAPK–ADP–Mg^2+^ and DAPK–AMP-PNP–Mg^2+^ complexes were determined at 1.85 and 2.00 Å resolution, respectively. Comparison of the two nucleotide-bound states with apo DAPK revealed localized changes in the glycine-rich loop region that were indicative of a transition from a more open state to a more closed state on binding of the nucleotide substrate and to an intermediate state with the bound nucleotide product.

## 1. Introduction

Death-associated protein kinase (DAPK) is a pro-apoptotic Ca^2+^/calmodulin-regulated serine/threonine protein kinase (Cohen *et al.*, 1997 ▶) that is a drug-discovery target for acute central nervous system (CNS) injuries (Schumacher, Velentza & Watterson, 2002 ▶; Velentza *et al.*, 2003 ▶; Craft *et al.*, 2004 ▶; Shamloo *et al.*, 2005 ▶). Acute CNS injuries such as stroke and traumatic brain injury are major causes of death, but the larger societal impact is subsequent morbidity, with life-long com­promised functioning among most survivors. Related to the latter point, single-nucleotide polymorphisms (SNPs) in the human DAPK gene have recently been shown to correlate with susceptibility to age-onset Alzheimer’s disease (Li *et al.*, 2006 ▶). The major impact of these disorders on health and morbidity and the lack of any disease-altering therapies have brought about a high level of scientific interest in the structure and function of DAPK.

Insight into the *in vivo* mechanisms by which DAPK could be involved in CNS pathophysiology (Schumacher, Velentza, Watterson *et al.*, 2002 ▶; Velentza *et al.*, 2003 ▶; Craft *et al.*, 2004 ▶; Shamloo *et al.*, 2005 ▶) has been provided by the identification (Schumacher *et al.*, 2004 ▶, 2006 ▶) of endogenous CNS protein substrates for DAPK. For example, the phosphorylation and inactivation of a downstream calmodulin-regulated protein kinase involved in neuronal survival by DAPK (Schumacher *et al.*, 2004 ▶) and the demonstration that DAPK is an S6 kinase that could alter neuronal cell protein biosynthesis (Schumacher *et al.*, 2006 ▶) raise the possibility that DAPK is involved in phosphorylation cascades that modulate the neuronal dysfunction or programmed cell death brought about by stress or injury. The participation of DAPK as an early step in an intracellular protein phosphorylation cascade before the cell is committed to death provides one possible explanation of how a single administration of a bioavailable DAPK inhibitor (Velentza *et al.*, 2003 ▶) in an early clinically relevant post-injury therapeutic time window can lead to the subsequent observation of im­proved neurologic outcomes (Velentza *et al.*, 2003 ▶; Shamloo *et al.*, 2005 ▶). Overall, *in vivo* studies and biochemical investigations have established the importance of the DAPK catalytic domain in pathophysiology progression and its potential as a therapeutic target.

Insight into the structural basis of the catalytic activity of DAPK was initially obtained by determination of the high-resolution crystal structures of the constitutively active DAPK catalytic domain in an apo form and in various complexes containing the ATP analog AMP-PNP (Tereshko *et al.*, 2001 ▶), combined with molecular model building and activity analyses of peptide-substrate preferences (Velentza *et al.*, 2001 ▶). Although crystallographic data are static representations of an average structure, the accumulation of structures of various complexes can provide insights related to the catalytic cycle. It has been noted (Nolen *et al.*, 2003 ▶) that examples of direct comparisons of protein kinase structures in the ADP-bound and AMP-PNP-bound forms, which reflect nucleotide product and substrate complexes, respectively, are limited. While additional serine/threonine protein kinase catalytic domain structures (Aoki *et al.*, 2004 ▶) and various complexes (Yang *et al.*, 2004 ▶) have been reported more recently, examples for the calmodulin-regulated protein kinase family are lacking. Therefore, we determined the structure of the DAPK–ADP–Mg^2+^ complex, based on the rationale that release of ADP is a rate-limiting step in protein kinase-mediated catalytic cycles (Adams, 2001 ▶), and compared its structure with that con­taining the hydrolysis-resistant ATP analog AMP-PNP and Mg^2+^ as well as with that of the apo form of DAPK.

Our results presented here provide the first DAPK–ADP–Mg^2+^ structure and the comparisons among different conformations reveal apparent nucleotide-induced changes in the DAPK structure that are localized to the glycine-rich loop region. The glycine-rich loop is a conserved region of protein kinases that has been proposed to be essential for catalysis (Johnson *et al.*, 2001 ▶) and is described by the consensus sequence -Gly-*X*-Gly-*X*-*X*-Gly. The first and second glycines occur in 95 and 99% of kinases, respectively, while the third glycine is conserved in 85% of kinases (Hemmer *et al.*, 1997 ▶). DAPK is among the 15% of kinases that lack the third glycine. The results obtained in this study allow a comparative analysis across the three conformations of DAPK.

## 2. Materials and methods

### 2.1. Enzyme preparation

The constitutively active catalytic domain of human DAPK, consisting of amino acids 1–285 of the cDNA open reading frame, was expressed and purified as described previously (Velentza *et al.*, 2001 ▶), except for the anion-exchange chromatography step. Briefly, bacterial cultures were grown in Luria broth at 310 K until the OD_600_ reached 0.6 and recombinant DNA-encoded expression of DAPK protein was in­duced by the addition of 0.2 mg anhydrotetracycline per litre of culture. Cultures were grown for an additional 3 h at 310 K and cell pellets were then collected by centrifugation at 5100*g* for 20 min and stored at 253 K. Rapidly thawed cell pellets were subjected to sonication in 100 m*M* Tris pH 8.0, 0.25 *M* NaCl, 0.01% Triton X-100, 0.7 mg l^−1^ pepstatin A and 1 m*M* DTT and then centrifuged at 39 000*g* for 40 min. The supernatant was collected, loaded onto 5 ml Streptactin affinity resin (Qiagen, Valencia, California, USA) and washed with 100 m*M* Tris–HCl pH 8.0, 0.25 *M* NaCl; DAPK was eluted with a buffer containing 2.5 m*M* desthiobiotin, 100 m*M* Tris–HCl pH 8.0, 0.25 *M* NaCl. The sample was concentrated at 277 K using 10 000 molecular-weight cutoff concentrators (Vivascience/Sartorius, Aubagne, France) at 5000*g* for 30 min and the buffer was exchanged for a buffer consisting of 0.25 *M* NaCl and 100 m*M* Tris pH 8.0. Purification of human DAPK for structures in the absence of Mg^2+^ was performed as described previously (Velentza *et al.*, 2001 ▶). The protein con­centration was determined by UV absorbance at 280 nm using a NanoDrop system (Wilmington, Delaware, USA) and a molar extinction coefficient of 92.7 g^−1^ ml cm^−1^. The protein was homogenous by SDS–PAGE and gave the expected molecular chain weight.

### 2.2. Crystallization and data collection

For the complex of DAPK with ADP (Sigma catalog No. A5410; lot 50H7080) and Mg^2+^, a protein solution at 5.5 mg ml^−1^ in 100 m*M* Tris pH 8.0 and 0.25 *M* NaCl was incubated with 1 m*M* ADP and 10 m*M* MgCl_2_ for 45 min at 277 K. Crystallization conditions were determined with 96-­well plate NeXtal AmSO4 suite screens (catalog No. 130905; Qiagen, Valencia, California, USA) using the sitting-drop method for crystallization. Crystals grew in 0.2 *M* ammonium chloride, 2.2 *M* ammonium sulfate pH 5.27 at 295 K and were harvested, soaked in a cryoprotection solution consisting of 0.2 *M* ammonium chloride, 2.2 *M* ammonium sulfate, 38% sucrose, 50 m*M* MgCl_2_ and 2.8 m*M* ADP and flash-frozen in liquid nitrogen. Diffraction data were collected using an MX-­300 detector (Rayonix LLC, Evanston, Illinois, USA) at 100 K on beamline 21-ID-G (LS-CAT) at the Advanced Photon Source (APS).

For the complex of DAPK with AMP-PNP (Sigma catalog No. A2647; lot 048 K5051) and Mg^2+^, a protein solution at 5.5 mg ml^−1^ in 100 m*M* Tris pH 8.0, 0.25 *M* NaCl was incubated with 1 m*M* AMP-PNP and 10 m*M* MgCl_2_ for 45 min at 277 K. Crystallization conditions for DAPK–AMP-PNP–Mg^2+^ were determined analogously to the DAPK–ADP–Mg^2+^ con­ditions using AmSO4 suite screens. Crystals grew in 0.2 *M* NaCl, 2.2 *M* ammonium sulfate pH 5.25 at 295 K and were harvested, soaked in a cryoprotection solution consisting of 0.2 *M* NaCl, 2.2 *M* ammonium sulfate, 30% sucrose, 6.0 m*M* AMP-PNP and 50 m*M* MgCl_2_ and flash-frozen in liquid nitrogen. Diffraction data were collected using an MX-300 detector (Rayonix LLC, Evanston, Illinois, USA) at 100 K on beamline 21-ID-G (LS-CAT) at the APS.

### 2.3. Structure determination and refinement

The data sets were integrated and merged using *HKL*-2000 (Otwinowski & Minor, 1997 ▶). Both nucleotide complexes belonged to the orthorhombic space group *P*2_1_2_1_2_1_, with unit-cell parameters *a* = 46.8, *b* = 62.6, *c* = 88.4 Å for DAPK–ADP–Mg^2+^ and *a* = 46.7, *b* = 62.5, *c* = 88.6 Å for DAPK–AMP-PNP–Mg^2+^. Both structures were determined by molecular replace­ment using *MOLREP* (Vagin & Teplyakov, 1997 ▶); apo DAPK with waters removed at 1.5 Å resolution (PDB code 1jks) was used as the search model. Interactive rounds of model building were performed in *Coot* (Emsley & Cowtan, 2004 ▶), refinement was performed with *REFMAC*5 (Mur­shudov *et al.*, 1997 ▶) and solvent was added using *ARP*/*wARP* (Perrakis *et al.*, 1997 ▶). The DAPK–ADP–Mg^2+^ structure (PDB code 3f5g) had a final crystallographic *R* factor and *R*_free_ factor of 0.19 and 0.24, respectively. The r.m.s. deviations from the ideal values were 0.015 Å for bond lengths and 1.57° for bond angles. The DAPK–AMP-PNP–Mg^2+^ structure (PDB code 3f5u) had a final crystallographic *R* factor and *R*_free_ factor of 0.18 and 0.25, respectively. The r.m.s. deviations from the ideal values were 0.018 Å for bond lengths and 1.77° for bond angles. Data-collection and refinement statistics are shown in Table 1 ▶. The structures of the nucleotide complexes in the absence of Mg^2+^ were determined for the purpose of determining the structural contribution of Mg^2+^ to the conformational differences observed on superposition. The DAPK–ADP com­plex (PDB code 3eh9) diffracted to 1.70 Å resolution and the DAPK–AMP-­PNP complex (PDB code 3eha) diffracted to 1.60 Å resolution; both were isomorphous to the other DAPK structures. Superpositions of protein structures were calculated over all atoms using least-squares fitting within *Coot* (Emsley & Cowtan, 2004 ▶). Figures were prepared using *PyMOL* (DeLano, 2002 ▶).

## 3. Results and discussion

### 3.1. Structure of DAPK–ADP–Mg^2+^ (PDB code 3f5g)

The structure of DAPK complexed with ADP is important because the release of ADP is a rate-limiting step in the kinase-mediated catalytic cycle (Adams, 2001 ▶). Our goal was to determine how the recognition of ADP by DAPK induces changes in the positioning of residues relative to other DAPK structures. We used cocrystallization of DAPK with ADP and Mg^2+^ followed by soaking of DAPK crystals with ADP and Mg^2+^ in a cryoprotection solvent. Crystallizations were performed under ammonium sulfate conditions similar to those described previously (Tereshko *et al.*, 2001 ▶; Velentza *et al.*, 2003 ▶). The crystals diffracted to 1.85 Å resolution and were isomorphous to previously determined DAPK structures.

**Figure 1:**
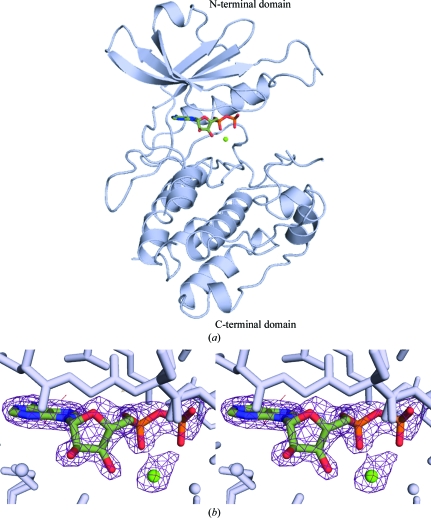
The structure of DAPK–ADP–Mg^2+^. (*a*) A representation of the overall structure of DAPK–ADP–Mg^2+^. (*b*) An *F*_o_ − *F*_c_ density map calculated with ADP and Mg^2+^ omitted from the model is shown contoured at +3σ within the context of the surrounding protein residues.

The structure of DAPK–ADP–Mg^2+^ (Fig. 1 ▶ *a*) shows that the structure has the canonical kinase fold consisting of a mainly N-terminal β-sheet and a larger α-helical C-terminal domain. The *F*_o_ − *F*_c_ OMIT map shows density corresponding to the ADP molecule between the N-terminal and C-­terminal domains (Fig. 1 ▶ *b*). Density for the adenine ring, the ribose ring, phosphates and one magnesium ion is clearly identifiable.

### 3.2. Superposition of DAPK–ADP–Mg^2+^ with apo DAPK

**Figure 2:**
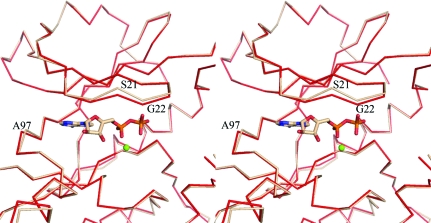
Superposition by least-squares fitting of DAPK–ADP–Mg^2+^ and apo DAPK reveals little change between the two structures apart from in two key areas: the hinge region near Ala97 and the glycine-rich loop near Ser21. The DAPK–ADP–Mg^2+^ structure is shown in red and the apo DAPK structure is shown in beige. Protein residues from the N-terminal domain and hinge region are shown.

A superposition of DAPK–ADP–Mg^2+^ and apo DAPK (PDB code 1jks; Tereshko *et al.*, 2001 ▶) was calculated by least-squares fitting (Emsley & Cowtan, 2004 ▶) over all atoms and an r.m.s. deviation of 0.69 Å was obtained. Overall, the structures of DAPK–ADP–Mg^2+^ and apo DAPK are mostly superim­posable, except for localized differences in the hinge and glycine-rich loop regions. The hinge region has a slight change in positioning that starts at the C^α^ atom of Val96 and continues through residue Ala97. In the superposition, the C^α^ atom of Ala97 in the DAPK–ADP–Mg^2+^ complex (PDB code 3f5g) differs by 0.85 Å from the position of  this atom in the apo DAPK structure (Fig. 2 ▶). The deviation in the glycine-rich loop of the DAPK structures begins at the C^α^ atom of Glu17 and continues through Phe24. The structure around Ser21 and Gly22 in DAPK–ADP–Mg^2+^ is apparently shifted towards the C-­terminal domain, resulting in a slight closing of the loop compared with the apo DAPK structure (Fig. 2 ▶). The amide N atom of Ser21 in the DAPK–ADP–Mg^2+^ structure differs by 1.10 Å from its position in the apo DAPK structure. The highly conserved Gly22 can be modeled in two conformations; the amide N of conformation *A* shown in Fig. 2 ▶ differs by 0.93 Å from the apo DAPK structure, while the amide N of conformation *B* (not shown in the figure) differs by 0.74 Å from the amide N of the apo DAPK structure.

### 3.3. Structure of DAPK–AMP-PNP–Mg^2+^ (PDB code 3f5u)

The structure of DAPK–ADP–Mg^2+^ was compared with the structures of DAPK–AMP-PNP–Mg^2+^ (PDB code 1jkk) and DAPK–AMP-PNP–Mn^2+^ (PDB code 1ig1) in order to examine conformational differences between the proteins. This was also performed to compare the detailed inter­actions of the protein with the nucleotide and the metal ion. However, the structures 1jkk and 1ig1 could not be used for com­parison with DAPK–ADP–Mg^2+^ owing to a lack of discernible density for the nucleotide β- and γ-phosphates. Therefore, we determined a new crystal structure of DAPK–AMP-PNP–Mg^2+^ using a combination of cocrystallization of DAPK with AMP-PNP and MgCl_2_ followed by soaking of crystals in a cryoprotection solution that contained AMP-PNP and MgCl_2_, similar to the method used to obtain the DAPK–ADP–Mg^2+^ complex. The DAPK–AMP-PNP–Mg^2+^ crystals grew in a high concentration of ammonium sulfate and diffracted to 2.00 Å resolution. The crystals were iso­morphous to previously determined DAPK structures. The data-collection and refinement statistics are shown in Table 1 ▶.

**Figure 3:**
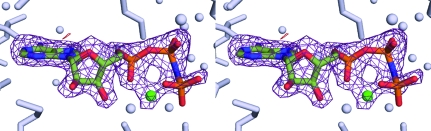
An *F*_o_ − *F*_c_ density map calculated with AMP-PNP and Mg^2+^ omitted from the model is shown contoured at +3σ within the context of the surrounding protein residues. The positions of the β- and γ-phosphates can be identified; however, the γ-phosphate has partial occupancy.

The structure of DAPK–AMP-PNP–Mg^2+^ (Fig. 3 ▶; PDB code 3f5u) was determined and the *F*_o_ − *F*_c_ OMIT map contained clear density for the nucleotide, including the β- and γ-phosphates and one magnesium ion. The positioning of the γ-phosphate in the new DAPK–AMP-PNP–Mg^2+^ structure is such that it is in a ‘kinked’ conformation similar to that observed in other kinase–AMP-PNP structures such as PKA (Kim *et al.*, 2005 ▶), JNK3 (Xie *et al.*, 1998 ▶), GSK3β (Aoki *et al.*, 2004 ▶), CDK2 (Brown *et al.*, 1999 ▶) and phosphorylase kinase (Lowe *et al.*, 1997 ▶). Initial modeling and refinement revealed partial negative density located around the γ-phosphate of the nucleotide, despite the use of the highest purity nucleotide available (see §[Sec sec2]2). However, this finding is not uncommon in kinase structures. For example, observation of negative density or a lack of density corresponding to the γ-­phosphate of AMP-PNP in a kinase complex can be seen in the electron-density maps of structures such as GSK3β–AMP-PNP (PDB code 1pyx), Polo-like kinase 1 (PDB code 2ou7), CK2–AMP-PNP (1lp4), Pim 1 kinase–AMP-PNP (PDB code 1yxt) and serum/glucocorticoid regulated kinase–AMP-PNP (PDB code 2r5t). Nevertheless, our new DAPK–AMP-PNP–Mg^2+^ data provided an improvement in density that allowed modeling of the γ-­phosphate. The adjustment of the occupancies of the γ-­phosphate and the OG1, O2G and O3G atoms to 0.60 resulted in elimination of the negative density in the refinement model, thereby allowing a comparative analysis between nucleotide-bound states.

### 3.4. Comparison of nucleotide and magnesium interactions in DAPK–ADP–Mg^2+^ and DAPK–AMP-PNP–Mg^2+^

**Figure 4:**
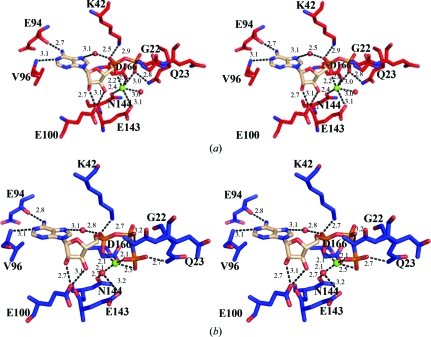
DAPK–nucleotide–Mg^2+^ interactions. (*a*) ADP–Mg^2+^ interactions with DAPK. (*b*) AMP-PNP–Mg^2+^ interactions with DAPK.

The determination of a DAPK–ADP–Mg^2+^ structure and a DAPK–AMP-PNP–Mg^2+^ structure with sufficient density to allow placement of the β- and γ-phosphates facilitated a comparison of the two protein conformations and examination of the bound nucleotides in the two DAPK structures. Superposition of DAPK–AMP-PNP–Mg^2+^ and DAPK–ADP–Mg^2+^ by a least-squares fit over all atoms revealed an r.m.s. deviation of 0.67 Å between the two structures. Examination of the details of nucleotide interactions in the DAPK–ADP–Mg^2+^ and DAPK–AMP-PNP–Mg^2+^ structures re­vealed con­servation of some key distances between the protein and the adenine and ribose portions of the nucleotides (Figs. 4 ▶ *a* and 4 ▶ *b*). For example, the adenine bases of AMP-PNP and ADP make conserved contacts with the hinge region of the kinase. The N1 atoms of the adenine rings are both 3.1 Å from the amide N atom of Val96. The N6 atom of the adenine ring is 2.7 Å away from the carboxyl of Glu94. The ribose-ring O2* and O3* atoms make conserved interactions with Glu100 in the ADP and AMP-PNP structures.

An examination of the protein–phosphate interactions revealed several contacts that are conserved among the ADP and AMP-PNP phosphates. The O2A atom of the α-phosphate in the ADP and AMP-PNP structures interacts with a water molecule and the NZ atom of the catalytic Lys42. The β-­phosphates of both structures are within proximity of the C^α^ atom of Gly22 of the glycine-rich loop. Several differences can be noted in the interactions of the β-phosphates. In the ADP structure the β-phosphate O3B atom is within proximity of the Mg^2+^ ion, while no β-phosphate atoms make contact with the Mg^2+^ ion of AMP-PNP. The improved density of the γ-phosphate of AMP-PNP allows a closer examination of protein atoms that are within hydrogen-bonding distance of the γ-­phosphate atoms and also those atoms within proximity that might be involved in the phospho-transfer process. The only protein atom that is within potential hydrogen-bonding distance is Asp139 OD1 (2.8 Å). Residues which are just beyond hydrogen-bonding distance to the γ-phosphate atoms but may aid in facilitating phospho-transfer include Asp139 (OD1 atom, 3.2 Å), Glu143 (OE2 atom, 4.0 Å), Gly22 (N atom, 4.4 Å), Gln23 (N atom, 3.7 Å) and Asn144 (ND2 atom, 4.4 Å).

In both the AMP-PNP-bound and ADP-bound forms, one magnesium ion is found in each structure. However, it cannot be ruled out that DAPK can bind two Mg^2+^ ions based on the current crystal structures. This is because the ammonium sulfate crystallization conditions may have competed with a weakly bound second Mg^2+^ ion. Most kinases will bind two magnesium ions (Adams, 2001 ▶). The first site (Mg1) involves the chelation of the β- and γ-phosphates with a conserved Asp. The second site (Mg2) is involved in chelation of the α- and γ-phosphates and is near a conserved Asp and a conserved Asn. The exact roles of the magnesium ions in catalysis can differ between kinases. In PKA, the first site is activating and the second site is inhibitory (Adams, 2001 ▶). It has been noted (Nolen *et al.*, 2003 ▶) that CDK2, casein kinase 1 and CK2α are examples of active protein kinases with bound ATP or ADP which bind only one Mg^2+^ ion in the Mg2 site. DAPK is similar to other kinases in that it binds one Mg^2+^ ion in the Mg2 site in the AMP-PNP-bound and ADP-bound forms, which may be the higher affinity coordination site.

**Figure 5:**
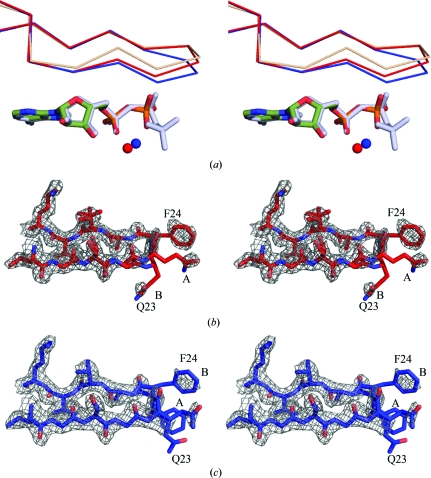
Comparisons of the glycine-rich loop. (*a*) Superposition of the glycine-rich loops of apo DAPK (beige), DAPK–ADP–Mg^2+^ (red) and DAPK–AMP-PNP–Mg^2+^ (blue) and their position relative to the nucleotide. The DAPK–AMP-PNP–Mg^2+^ loop has the most closed con­formation. Residues 17–28 are shown for simplicity. The calculated r.m.s. deviation over the C^α^ atoms of residues 20–25 between the apo DAPK structure and the DAPK–AMP-PNP–Mg^2+^ structure is 1.03 Å and that between the apo DAPK and DAPK–ADP–Mg^2+^ structures is 0.62 Å. (*b*) 2*F*_o_ − *F*_c_ electron-density map at 1.0σ of the glycine-rich loop of DAPK–ADP–Mg^2+^. One conformation of Gln23 is modeled such that the side chain is within proximity of the β-phosphate (conformation *B*). (*c*) 2*F*_o_ − *F*_c_ electron-density map of the glycine-rich loop of DAPK–AMP-PNP–Mg^2+^. The side chain of Phe24 in the DAPK–AMP-PNP structure can be modeled in the ‘open’ conformation (*B*) or a conformationally restricted position (*A*).

In a superposition of the ADP and AMP-PNP structures, the location of the magnesium ion differs by 1.2 Å (Fig. 5 ▶ *a*). The Mg^2+^ ion in the ADP structure is coordinated to six O atoms, which include the O1A atom of the α-phosphate, the O3B atom of the β-phosphate, a water molecule, Glu143 OE2, Asn144 OD1 and Asp161 OD1. In the AMP-PNP structure, the Mg^2+^ ion makes fewer interactions with the protein residues and only coordinates to Asp161 OD2, while other inter­actions are made with the O1A atom of the α-­phosphate, the O3G and O2G atoms of the γ-phosphate and a water molecule. An inspection of the crystal structure of an ADP complex obtained in the absence of Mg^2+^, DAPK–ADP (PDB code 3eh9), reveals an alternative conformation of the β-­phosphate. In this alternative conformation, the phosphate is in a similar location to the position where Mg^2+^ binds. This raises the possibility that the role of Mg^2+^ is to assist in the selection of a catalytically preferred conformation of the β-­phosphate. In the AMP-PNP structure obtained in the absence of Mg^2+^, DAPK–AMP-PNP (PDB code 3eha), only subtle changes in interaction distances are seen between the diaxial O atoms of the β- and γ-phosphates and the glycine-rich loop. For example, in the absence of Mg^2+^, the amide N interaction distances to the diaxial O atom of the β-phosphate are 3.3 Å to Ala25 and 2.9 Å to Asn24. In the Mg^2+^ structure, these distances are increased to 3.8 and 4.4 Å, respectively.

### 3.5. Comparison of the glycine-rich loop conformations of DAPK structures

A comparison of the apo DAPK, DAPK–ADP–Mg^2+^ and DAPK–AMP-PNP–Mg^2+^ structures revealed an interesting structural change in the glycine-rich loop region. Similar to the comparisons of DAPK–ADP–Mg^2+^ with the apo and AMP-PNP- and Mg^2+^-bound forms, a superposition of the DAPK–AMP-PNP–Mg^2+^ structure and the apo DAPK structure (PDB code 1jks) was calculated so that a comparison of r.m.s deviation values could be generated among all three structures. The r.m.s. deviation was 0.59 Å, which is comparable to the other superpositions. Although all three structures generally superimposed, differences were noted in the topology of the glycine-rich loop encompassing amino acids 20–25 (Fig. 5 ▶ *a*). Comparatively, the DAPK–AMP-PNP–Mg^2+^ structure is a more closed structure, the apo DAPK (PDB code 1jks) structure is a more open structure and the DAPK–ADP–Mg^2+^ structure is intermediate between the two.

The variance of the glycine-rich loop conformations relative to the apo DAPK structure is reflected in the r.m.s. deviation values calculated over the C^α^ atoms for residues 20–25. The r.m.s. deviation for these residues between apo DAPK and DAPK–AMP-PNP–Mg^2+^ is 1.03 Å and that between apo DAPK and DAPK–ADP–Mg^2+^ is 0.62 Å. A closed conformation of the DAPK–AMP-PNP–Mg^2+^ and DAPK–ADP–Mg^2+^ structures indicates that the loop is more ordered, or conformationally restricted, by the phosphate interactions. Stabilization of the glycine-rich loop by an ATP analog is consistent with studies of other protein kinases (Xie *et al.*, 1998 ▶; Johnson *et al.*, 2001 ▶). An active-site opening and closing that involves changes in the glycine-rich loop conformation would be consistent with the prevailing model of conformational flexibility being essential for catalysis (Johnson *et al.*, 2001 ▶). However, the observation that AMP-PNP takes the most closed conformation has not always been observed, as in the case of complexes of the serine-specific kinase Sky1p (Nolen *et al.*, 2003 ▶). The trend of a comparatively open and flexible conformation for DAPK–ADP–Mg^2+^ compared with DAPK–AMP-PNP–Mg^2+^ might be related to the release of the reaction products ADP and the phosphorylated peptide.

Several assumptions have been made in modeling side chains in this region that deserve mention. Firstly, Gln23 in the glycine-rich loop region is modeled in two different conformations in the DAPK–ADP–Mg^2+^ structure and in two different conformations in the DAPK–AMP-PNP–Mg^2+^ structure (Figs. 5 ▶ *b* and 5 ▶ *c*). Interestingly, a possible stabilizing role of the Gln23 side chain in the ADP structure is suggested by the 2.8 Å distance between Gln23 NE2 and the O3B atom of the β-phosphate (Fig. 4 ▶ *a*). The position of Gln23 NE2 occupies the same location as the position of the γ-phosphate in the AMP-PNP structure. In the AMP-PNP structure, the NE2 atom is modeled in a position that is within proximity of the γ-­phosphate; however, at 1.0σ density can only be seen for the CA and CB atoms for this conformation, so it is unclear whether Gln23 NE2 is involved in an interaction with the γ-­phosphate O atoms (Fig. 5 ▶ *c*). The results raise the possibility that Gln23 might be important in the catalytic cycle owing to its potential hydrogen-bonding capability with the β-phosphate of ADP and possibly the γ-phosphate of AMP-PNP, thereby contributing to the conformational adaptability that is key to catalysis.

Secondly, Phe24 also appears to have conformational variability. The conformation of the side chain of Phe24 in apo DAPK and DAPK–ADP–Mg^2+^ is up towards the N-terminal domain. In the AMP-PNP-bound form, an alternative conformation of Phe24 with the side chain down towards the C-terminal domain can be modeled in a conformation distinct from the conformation in DAPK–ADP and apo DAPK (Fig. 5 ▶ *c*). The two conformations result in a 1.6 Å difference in the C^α^ atom of Phe24.

The three DAPK conformations demonstrate that nucleotide binding brings about changes in conformation that are mostly localized to the glycine-rich loop region. This trend is consistent with the results for other protein kinases (Yang *et al.*, 2004 ▶; Xie *et al.*, 1998 ▶; Nolen *et al.*, 2003 ▶; Eswaran *et al.*, 2007 ▶; Lowe *et al.*, 1997 ▶). Interestingly, Gln23 within the glycine-rich loop may play a role in regulating ADP binding/release, which is the rate-limiting step in the catalytic mechanism. The mutagenesis of this residue and an examination of the effects on catalytic activity would be an interesting test of the hypothesis in future studies.

## 4. Conclusions

The determination of the first DAPK–ADP–Mg^2+^ structure and the determination of a new DAPK–AMP-PNP–Mg^2+^ structure allowed a comparative analysis of three different DAPK conformations relevant to the catalytic cycle: a form containing no bound nucleotide, a form containing a non­hydrolyzable analog of the ATP sub­strate and a form containing the reaction product ADP. The comparisons revealed discrete localized conformational changes in DAPK as a result of changes in nucleotide binding. The most notable conformational changes occur in the glycine-rich loop region, an active-site region with potential for modulation of kinase activity.

## Supplementary Material

PDB reference: DAPK–ADP–Mg^2+^, 3f5g, r3f5gsf

PDB reference: DAPK–AMP-PNP–Mg^2+^, 3f5u, r3f5usf

PDB reference: DAPK–ADP, 3eh9, r3eh9sf

PDB reference: DAPK–AMP-PNP, 3eha, r3ehasf

## Figures and Tables

**Table 1:** Data-collection and refinement statistics Values in parentheses are for the highest resolution shell.

	DAPK–ADP–Mg^2+^	DAPK–AMP-PNP–Mg^2+^
Residue boundaries	1–285	1–285
Ligand	ADP–Mg^2+^	AMP-PNP–Mg^2+^
PDB code	3f5g	3f5u
Space group	*P*2_1_2_1_2_1_	*P*2_1_2_1_2_1_
Unit-cell parameters (Å)	*a* = 46.8, *b* = 62.6, *c* = 88.4	*a* = 46.7, *b* = 62.5, *c* = 88.6
X-ray source	21-ID-G (LS-CAT)	21-ID-G (LS-CAT)
Wavelength (Å)	0.97857	0.97857
Resolution (Å)	30.0–1.85 (1.92–1.85)	30.0–2.0 (2.07–2.00)
No. of observations	159531	126280
No. of unique reflections	22396 (2154)	18512 (1132)
Cutoff criterion	*I* < −3σ(*I*)	*I* < −3σ(*I*)
Completeness (%)	98.08 (96.26)	99.05 (87.70)
Redundancy	3.8 (3.8)	6.8 (6.6)
Mean *I*/σ(*I*)	10.6 (4.15)	9.10 (3.20)
*R*_merge_† (%)	0.066 (0.469)	0.068 (0.546)
Wilson *B* factor (Å^2^)	32.0	36.2
Model and refinement statistics		
Resolution range (Å)	30.0–1.85	30.0–2.00
No. of reflections	21206	17521
Final *R*_work_‡	0.185 (0.197)	0.177 (0.199)
No. of reflections in test set for *R*_free_§	1147	941
Final *R*_free_§	0.244 (0.257)	0.245 (0.338)
R.m.s. deviation from ideal		
Bond lengths (Å)	0.015	0.018
Bond angles (°)	1.57	1.77
Average *B* factors (Å^2^)	25.7	30.3
Ramachandran plot analysis		
Most favored regions (%)	90.4	91.5
Additionally allowed regions (%)	9.2	8.1
Generously allowed regions (%)	0.4	0.4
Disallowed regions (%)	0.0	0.0

† *R*_merge_ = 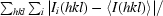 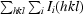.

‡ *R*_work_ = 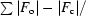 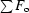.

§ 5% of the reflections were reserved for calculation of *R*_free_.

## References

[bb1] Adams, J. A. (2001). *Chem. Rev.* **101**, 2271–2290. 10.1021/cr000230w 11749373

[bb2] Aoki, M., Yokota, T., Sugiura, I., Sasaki, C., Hasegawa, T., Okumura, C., Ishiguro, K., Kohno, T., Sugio, S. & Matsuzaki, T. (2004). *Acta Cryst.* D**60**, 439–446. 10.1107/S090744490302938X 14993667

[bb3] Brown, N. R., Noble, M. E. M., Endicott, J. A. & Johnson, L. N. (1999). *Nature Cell. Biol.* **1**, 438–443. 10.1038/15674 10559988

[bb5] Cohen, O., Feinstein, E. & Kimchi, A. (1997). *EMBO J.* **15**, 998–1008. 10.1093/emboj/16.5.998 PMC1169700 9118961

[bb4] Craft, J. M., Watterson, D. M., Frautschy, S. A. & Van Eldik, L. J. (2004). *Neurobiol. Aging*, **25**, 1283–1292. 10.1016/j.neurobiolaging.2004.01.006 15465624

[bb6] DeLano, W. L. (2002). *The PyMOL Molecular Graphics System.* DeLano Scientific, Palo Alto, California, USA.

[bb7] Emsley, P. & Cowtan, K. (2004). *Acta Cryst.* D**60**, 2126–2132. 10.1107/S0907444904019158 15572765

[bb8] Eswaran, J., Lee, W. H., Debreczeni, J. É., Filippakopoulos, P., Turnbull, A., Federov, O., Deacon, S. W., Peterson, J. R. & Knapp, S. (2007). *Structure*, **15**, 201–213. 10.1016/j.str.2007.01.001 PMC1885963 17292838

[bb9] Hemmer, W., McGlones, M., Tsigelny, I. & Taylor, S. S. (1997). *J. Biol. Chem.* **272**, 16946–16954. 10.1074/jbc.272.27.16946 9202006

[bb10] Johnson, D. A., Akamine, P., Radzio-Andzelm, E., Madhusudan & Taylor, S. S. (2001). *Chem. Rev.* **101**, 2243–2270. 10.1021/cr000226k 11749372

[bb11] Kim, C., Xuong, N.-H. & Taylor, S. S. (2005). *Science*, **307**, 690–696. 10.1126/science.1104607 15692043

[bb12] Li, Y. *et al.* (2006). *Hum. Mol. Genet.* **15**, 2560–2568. 10.1093/hmg/ddl178 16847012

[bb13] Lowe, E. D., Noble, M. E. M., Skamnaki, V. T., Oikonomakos, N. G., Owen, D. J. & Johnson, L. N. (1997). *EMBO J.* **16**, 6646–6658. 10.1093/emboj/16.22.6646 PMC1170269 9362479

[bb14] Murshudov, G. N., Vagin, A. A. & Dodson, E. J. (1997). *Acta Cryst.* D**53**, 240–255. 10.1107/S0907444996012255 15299926

[bb15] Nolen, B., Ngo, J., Chakrabarti, S., Vu, D., Adams, J. A. & Ghosh, G. (2003). *Biochemistry*, **42**, 9575–9585. 10.1021/bi0344331 12911299

[bb16] Otwinowski, Z. & Minor, W. (1997). *Methods Enzymol.* **276**, 307–326. 10.1016/S0076-6879(97)76066-X 27754618

[bb17] Perrakis, A., Sixma, T. K., Wilson, K. S. & Lamzin, V. S. (1997). *Acta Cryst.* D**53**, 448–455. 10.1107/S0907444997005696 15299911

[bb18] Schumacher, A. M., Schavocky, J. P., Velentza, A. V., Mirzoeva, S. & Watterson, D. M. (2004). *Biochemistry*, **43**, 8116–8124. 10.1021/bi049589v 15209507

[bb19] Schumacher, A. M., Velentza, A. V. & Watterson, D. M. (2002). *Expert Opin. Ther. Targets*, **6**, 497–506. 10.1517/14728222.6.4.497 12223064

[bb20] Schumacher, A. M., Velentza, A. V., Watterson, D. M. & Dresios, J. (2006). *Biochemistry*, **45**, 13614–13621. 10.1021/bi060413y PMC4404312 17087515

[bb21] Schumacher, A. M., Velentza, A. V., Watterson, D. M. & Wainwright, M. S. (2002). *Biochim. Biophys. Acta*, **1600**, 128–137. 10.1016/s1570-9639(02)00453-3 12445468

[bb22] Shamloo, M., Soriano, L., Wieloch, T., Nikolich, K., Urfer, R. & Oksenberg, D. (2005). *J. Biol. Chem.* **280**, 42290–42299. 10.1074/jbc.M505804200 16204252

[bb23] Tereshko, V., Teplova, M., Brunzelle, J., Watterson, D. M. & Egli, M. (2001). *Nature Struct. Biol.* **8**, 899–907. 10.1038/nsb1001-899 11573098

[bb24] Vagin, A. & Teplyakov, A. (1997). *J. Appl. Cryst.* **30**, 1022–1025.

[bb25] Velentza, A. V., Schumacher, A. M., Weiss, C., Egli, M. & Watterson, D. M. (2001). *J. Biol. Chem.* **276**, 38956–38965. 10.1074/jbc.M104273200 11483604

[bb26] Velentza, A. V., Wainwright, M. S., Zasadzki, M., Mirzoeva, S., Schumacher, A. M., Haiech, J., Focia, P. J., Egli, M. & Watterson, D. M. (2003). *Bioorg. Med. Chem. Lett.* **13**, 3465–3470. 10.1016/s0960-894x(03)00733-9 14505650

[bb27] Xie, X., Gu, Y., Fox, T., Coll, J. T., Fleming, M. A., Markland, W., Caron, P. R., Wilson, K. P. & Su, M. S.-S. (1998). *Structure*, **6**, 983–991. 10.1016/s0969-2126(98)00100-2 9739089

[bb28] Yang, J., Ten Eyck, L. F., Xuong, N.-H. & Taylor, S. S. (2004). *J. Mol. Biol.* **336**, 473–487. 10.1016/j.jmb.2003.11.044 14757059

